# Synthesis of the 3’-*O*-sulfated TF antigen with a TEG-N_3_ linker for glycodendrimersomes preparation to study lectin binding

**DOI:** 10.3762/bjoc.20.17

**Published:** 2024-01-30

**Authors:** Mark Reihill, Hanyue Ma, Dennis Bengtsson, Stefan Oscarson

**Affiliations:** 1 Centre for Synthesis and Chemical Biology, University College Dublin, Belfield, Dublin 4, Ireland

**Keywords:** regioselective sulfation, thioglycoside donors, Thomsen–Friedenreich antigen

## Abstract

The synthesis of gram quantities of the TF antigen (β-ᴅ-Gal-(1→3)-α-ᴅ-GalNAc) and its 3’-sulfated analogue with a TEG-N_3_ spacer attached is described. The synthesis of the TF antigen comprises seven steps, from a known *N*-Troc-protected galactosamine donor, with an overall yield of 31%. Both the spacer (85%) and the galactose moiety (79%) were introduced using thioglycoside donors in NIS/AgOTf-promoted glycosylation reactions. The 3’-sulfate was finally introduced through tin activation in benzene/DMF followed by treatment with a sulfur trioxide–trimethylamine complex in a 66% yield.

## Introduction

**Figure 1:**
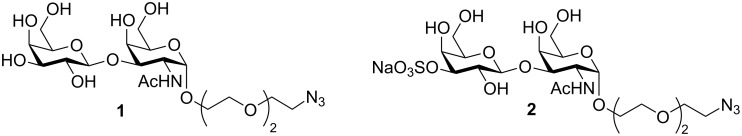
Structure of target compounds **1** and **2**.

In a collaboration project with groups from Universities in Munich and Pennsylvania we are investigating carbohydrate–lectin interactions using programmable glycodendrimersomes based on synthetic glycans. We have earlier synthesized 2-[2-(2-azidoethoxy)ethoxy]ethyl (TEG-N_3_) glycosides of lactose, 3’-Su-lactose and LacdiNAc (β-ᴅ-GalNAc-(1→4)-β-ᴅ-GlcNAc), which have then been used for production of the glycodenrimersomes and interaction studies with various galectins [1–2]. In the continuation of this collaboration, to investigate the binding of siglec-1 and the chimera of 3’-SuTF-binding siglecs and TF-binding galectin-3, TEG-N_3_ glycosides of the TF antigen (β-ᴅ-Gal-(1→3)-α-ᴅ-GalNAc, **1**) and its 3’-*O*-sulfated analogue (**2**, Figure 1) were required on a gram scale to allow efficient synthesis of the glycodendrisomes. The TF antigen is presented on the surface of most human cancer cell types and its interaction with galectins 1 and 3 leads to tumour cell aggregation and promotes cancer metastasis [3–5]. The 3’-*O*-sulfated analogue is known to bind to siglecs 1, 4, and 8 [6] as well as galectin 4 [7–8], but its biological role is not that well investigated.

Compound **2** is a new compound but two syntheses of compound **1** have recently been reported, one using an enzymatic approach and a commercial α-TEG-N_3_ GalNAc acceptor [9] and one using glycosyl bromide donors and silver salt-promoted glycosylations [10].

## Results and Discussion

**Scheme 1:**
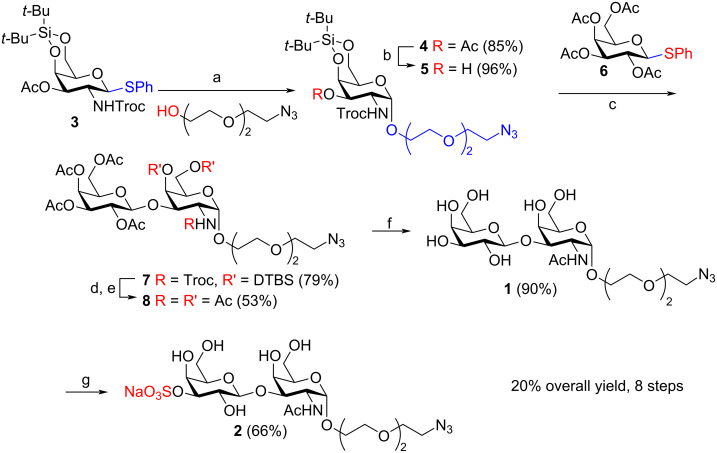
Synthesis of target compounds **1** and **2**. Key: a) NIS, AgOTf (20 mol %), 4 Å molecular sieves, CH_2_Cl_2_, rt, 40 min, 85%; b) NaOMe (10 mol %), MeOH, rt, 4 h, 96%; c) NIS, AgOTf (19 mol %), AW-300 4 Å molecular sieves, CH_2_Cl_2_, rt, 1 h, 79%; d) 1 M Bu_4_NF/THF, THF, rt, 2 h, then HF·Py, rt, 3 h; e) Ac_2_O/Py (1:2, v/v), rt, 16 h, 53% over 3 steps; f) 1 M NaOMe/MeOH, MeOH, pH 10, rt, 1 h, 90%; g) Bu_2_SnO, benzene/DMF (5:1, v/v), 125 °C, 24 h, then SO_3_-NMe_3_, DMF, rt, 72 h, then flash chromatography, then Dowex^®^ 50WX4 (Na^+^ form) resin, H_2_O, rt, 16 h, 66%.

To introduce the 2-[2-(2-chloroethoxy)ethoxy]ethyl (TEG-Cl) spacer both a Fischer synthesis starting from unprotected *N*-acetylgalactosamine and a Lewis acid-promoted reaction starting from per-acetylated galactosamine were initially tested. As reported [11], the Fischer synthesis gives low yields and α-selectivity. The Lewis acid-promoted reaction, which had worked well to produce β-linked TEG-spacer glycosides with per-acetylated lactose and 2-phthalimidoglucosamine [1–2] worked well with 2-chloroethanol as a spacer (68%, pure α) but failed with the TEG-Cl spacer [12], why we instead decided to use a thioglycoside donor to introduce the spacer. To ensure α-selectivity a di-*tert*-butylsilyl-4,6-acetal-protected donor, as developed by the Kiso group [13–14], was chosen. After some initial testing the known *N*-Troc-protected donor **3** [15–16] (Scheme 1) was selected [17].

Since donor **3** possessed a Troc group, which contains 3 chlorine atoms, nucleophilic introduction of an azido group at this stage was predicted to be problematic. Therefore, the azido functionality was installed in the spacer before the glycosylation. Donor **3** underwent an NIS/AgOTf-promoted glycosylation with the TEG-N_3_ acceptor [18], furnishing α-linked **4** in an 85% yield (Scheme 1). H-1 appeared as a doublet at 4.95 ppm with a *J* value of 3.6 Hz in the ^1^H NMR spectrum proving the anomeric α-configuration. The presence of Troc-rotamers was also apparent, with a ratio of 19:6 being observed by ^1^H NMR in CDCl_3_ at 25 °C. Catalytic amounts of NaOMe (0.005 M) in MeOH were used to remove the acetate from compound **4**, taking care not to affect the Troc group, to afford acceptor **5** in a 96% yield.

Earlier optimizations of the introduction of the β-linked galactose moiety using 2-azidoethyl 2-acetamido-4,6-*O*-benzylidene-2-deoxy-α-ᴅ-galactopyranoside as acceptor showed an acetylated thioglycoside donor to be the best choice [12], surprisingly better than a benzoylated donor [19], why this donor was the first one tested also with the quite different acceptor **5**. An NIS/AgOTf-promoted glycosylation with donor **6** [20] yielded 79% of disaccharide **7**. Due to the presence of rotamers, NMR spectra of **7** proved to be difficult to analyse when data were recorded in CDCl_3_. Changing the NMR solvent to CD_3_OD greatly reduced the complexity of the spectra [21–23].

Since **7** possessed an azido group as part of the linker, removal of the Troc group under reductive conditions was ruled out due to probable chemoselectivity issues [24–25]. Interestingly, Jacquemard et al*.* outlined a useful, mild method for removing a range of carbamates using Bu_4_NF in an article from 2004 [26]. As **7** contained a DTBS group, the possibility of removing both Troc and DTBS groups in a one-pot procedure was tested. Disaccharide **7** was therefore treated with 1 M Bu_4_NF/THF and after 2 hours, full consumption of the starting material was observed by TLC. However, MALDI–TOF mass spectrometry (super-DHB matrix) revealed that only the Troc group had been removed, with the DTBS substituent proving to be stable under these conditions. Addition of a large excess of HF·Py (40 equiv) proved to be necessary to remove the bulky silyl group. After concentration, the crude product was acetylated (Ac_2_O/Py, 1:2, v/v), furnishing per-acetylated compound **8** in a 53% yield over the 3 steps. Deacetylation of **8** with freshly prepared 1 M NaOMe/MeOH in MeOH at pH 10 furnished target **1** in a 90% yield.

Formation of a stannylidene acetal via tin-activation was employed to achieve selective 3’-*O*-sulfation of compound **1** [27], with a variety of conditions being attempted (Table 1). With a TEG-N_3_ lactose compound, tin-activation was performed with Bu_2_SnO in refluxing MeOH, followed by stirring with SO_3_·NMe_3_ in 1,4-dioxane to afford the 3’-*O*-sulfate in 65% yield [1]. Here, however, this choice of solvent in the sulfation step led to the material being insoluble and no reaction was observable by TLC. Changing the solvent of the sulfation reaction to DMF resulted in formation of a homogenous solution, but still no conversion to the sulfated product, even when the temperature was raised to 80 °C [28–29]. Switching the sulfating reagent to SO_3_·Py or performing the reaction at 150 °C in a microwave did not improve the outcome [30–31].

**Table 1:** Summary of conditions attempted to achieve regioselective 3’-*O*-sulfation.

Tin-activation^a^				Sulfation^a^			
		
Solvent(s)	Temperature	Set-up		Sulfating reagent	Solvent	Temperature/conditions	Result/ yield

MeOH	95 °C	reflux		SO_3_·NMe_3_	1,4-dioxane	rt	material insoluble, no reaction
MeOH	95 °C	reflux		SO_3_·NMe_3_	DMF	rt–80 °C	no reaction
MeOH	95 °C	reflux		SO_3_·Py	DMF	80 °C	no reaction
MeOH	95 °C	reflux		SO_3_·Py	DMF	150 °C, microwave	no reaction
benzene/DMF (5:1, v/v)	125 °C	reflux, Dean–Stark		SO_3_·NMe_3_	DMF	rt	66%

^a^Tin-activation was performed with 1.2 equiv of Bu_2_SnO in all cases for 16–24 h and sulfation reactions proceeded for 24–72 h.

**Figure 2:**
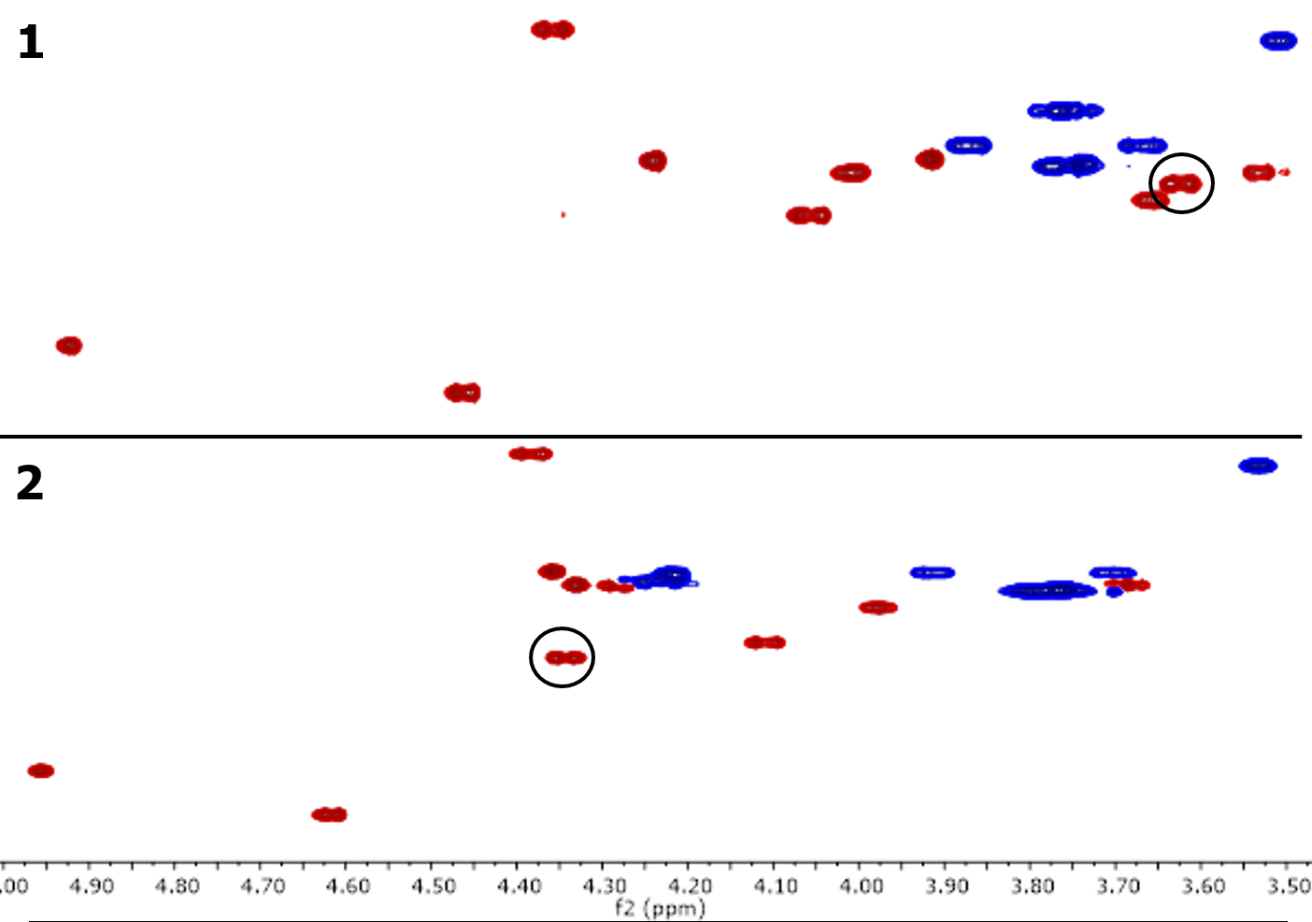
Comparison of the ^1^H,^13^C HSQC spectra of **1** (top) and 3’-*O*-sulfated **2** (bottom), with circles highlighting the signals corresponding to H-3’/C-3’.

Since there was no observable sulfation taking place, the tin-activation step was suspected to be the root of the problem. To rectify this, similar to Malleron et al., **1** was refluxed, in a Dean–Stark set-up, with Bu_2_SnO in benzene/DMF (5:1, v/v) [32]. The solvent in the receiver was drained after 24 hours and the benzene was removed from the reaction mixture in vacuo. Sulfation was then performed through addition of SO_3_·NMe_3_ to the DMF solution. Consumption of **1** was observed by TLC after 72 hours and stirring with Dowex^®^ 50WX4 (Na^+^ form) resin resulted in formation of target **2**. Purification by flash chromatography, however, led to isolation of a mixture of **2** and a tin-related impurity (*n*-butyl chain evident by NMR). Acetylation of this material followed by flash chromatography proved ineffective in removing the unwanted entity. To overcome this problem, flash chromatography was performed before stirring with the ion-exchange resin, with no apparent presence of tin impurities by NMR when the sequence was executed in this order and sulfated target **2** was obtained in a 66% yield on a one-gram scale. Comparing the ^1^H,^13^C HSQC spectra of compounds **1** and **2**, there is a clear downfield shift of the H-3’/C-3’ signal from **1** to sulfated **2** (Figure 2). This showed that regioselective 3’-*O*-sulfation had been achieved, with HRMS also indicating that only one sulfate group was present.

## Conclusion

An efficient synthesis of the important TF and 3’-Su-TF antigens equipped with a TEG-N_3_ linker to allow formation of various conjugates has been developed for further interaction studies with lectins (galectins and siglecs). The synthesis of the 3’-Su-TF antigen **2** comprises eight steps from the known *N*-galactosamine donor **3**, where two of the steps, removal of the Troc- and DTBS protecting groups are performed in the same pot and the following acetylation without purification of the intermediate, why the synthesis is high-yielding (20% overall yield) and easily scalable (9 g of protected disaccharide **7** and 1 gram of target **2** were synthesized).

## Experimental

### General methods

All reactions containing air- and moisture-sensitive reagents were carried out under an inert atmosphere of nitrogen in oven-dried glassware with magnetic stirring. N_2_-flushed plastic syringes were used to transfer air- and moisture-sensitive reagents. All reactions were monitored by thin-layer chromatography (TLC) on Merck^®^ DC-Alufolien plates precoated with silica gel 60 F_254_. Visualisation was performed with UV-light (254 nm) fluorescence quenching, and/or by staining with an 8% H_2_SO_4_ dip (stock solution: 8 mL conc. H_2_SO_4_, 92 mL EtOH), ninhydrin dip (stock solution: 0.3 g ninhydrin, 3 mL AcOH, 100 mL EtOH) and/or ceric ammonium molybdate dip (stock solution: 25 g ammonium molybdate tetrahydrate, 0.5 g Ce(SO_4_)_2_, 50 mL H_2_SO_4_, 450 mL EtOH).

### Chromatography

Silica gel flash chromatography was carried out using Davisil^®^ LC60A (40–63 μm) silica gel or with automated flash chromatography systems, Buchi Reveleris^®^ X2 (UV 200–500 nm and ELSD detection, Reveleris^®^ silica cartiges 40 μm, Büchi Labortechnik AG^®^) and Biotage^®^ SP4 HPFC (UV 200–500 nm, Biotage^®^ SNAP KP-Sil 50 μm irregular silica, Biotage^®^ AB).

### Instrumentation

^1^H NMR and ^13^C NMR spectra were recorded on Varian Inova spectrometers at 25 °C in chloroform-*d* (CDCl_3_), methanol-*d*_4_ (CD_3_OD), deuterium oxide (D_2_O) or DMSO-*d*_6_ ((CD_3_)_2_SO). ^1^H NMR spectra were standardised against the residual solvent peak (CDCl_3_, δ = 7.26 ppm; CD_3_OD, δ = 3.31 ppm; D_2_O, δ = 4.79 ppm; (CD_3_)_2_SO δ = 2.50 ppm); or internal trimethylsilane, δ = 0.00 ppm). ^13^C NMR spectra were standardised against the residual solvent peak (CDCl_3_, δ = 77.16 ppm; CD_3_OD, δ = 49.00 ppm; (CD_3_)_2_SO δ = 39.52 ppm and ^13^C NMR spectra recorded in D_2_O are unreferenced. All ^13^C NMR spectra are ^1^H decoupled. All NMR data are represented as follows: chemical shift (δ ppm), multiplicity (s = singlet, br s = broad singlet, d = doublet, app d = apparent doublet, t = triplet, q = quartet, dd = doublet of doublets, dt = doublet of triplets, m = multiplet), coupling constant in Hz, integration. Assignments were aided by homonuclear ^1^H,^1^H (COSY, TOCSY) and ^1^H,^13^C heteronuclear (HSQC, HMBC) two-dimensional correlation spectroscopies. ^13^C chemical shifts were reported to one decimal point unless an additional digit was required to distinguish overlapping peaks. Software for data processing: MestReNova, version 11.0.0–17609 (MestReLab Research S.L.). High-resolution mass spectrometry (HRMS) data were recorded on a Waters Micromass LCT LC–TOF instrument using electrospray ionisation (ESI) in positive mode. MALDI–TOF mass spectrometry data were recorded on a Scientific Analysis Instruments MALDI–TOF mass spectrometer in reflectron mode for oligosaccharides and in linear mode for glycoconjugates. Samples were prepared by pre-mixing 1 µL of a solution containing the analyte with 20 µL of a matrix solution (10 mg/mL, MeCN/H_2_O, 1:1, v/v + 1% TFA), pipetting 1 µL of the mixture onto the sample plate and drying under gentle heat from a heat gun. Optical rotations were recorded in a Perkin-Elmer polarimeter (Model 343) at the sodium D-line (589 nm) at 20 °C using a 1 dm cell. Samples were prepared at the concentration (g/100 mL) in the solvent indicated. Deprotected glycans were lyophilised using a freeze-dryer Alpha 1-2 LDplus (Christ Ltd): pressure: 0.055 mbar; ice-condenser temperature: −55 °C.

**2-[2-(2-Azidoethoxy)ethoxy]ethyl 3-*O*-acetyl-2-deoxy-4,6-*O*-di-*tert*-butylsilylene-2-(2’2’2’-trichloroethoxycarbonylamino)-α-ᴅ-galactopyranoside (4):** Donor **3** [9–10] (9.3 g, 15 mmol) and the TEG-N_3_ acceptor (synthesized as described in reference [12], but also commercially available, 3.9 g, 22 mmol) were placed under N_2_ together and dissolved in dry CH_2_Cl_2_ (300 mL). 4 Å molecular sieves (10.2 g) were added and the resulting suspension was stirred at room temperature for 16 hours. NIS (6.66 g, 29.6 mmol) and AgOTf (760 mg, 2.96 mmol) were then added, and the reaction was stirred at room temperature for 40 minutes. The reaction was then quenched with Et_3_N, filtered through Celite^®^ and concentrated in vacuo. The resulting residue was taken up in EtOAc (700 mL) and washed with 10% aq Na_2_S_2_O_3_ (700 mL), water (700 mL) and brine (700 mL). The organic phase was then dried over MgSO_4_, filtered and reduced to dryness. Flash chromatography on silica gel (toluene→toluene/EtOAc, 3:2) yielded **4** as an orange syrup (8.74 g, 85%). *R*_f_ = 0.4 (toluene/EtOAc, 7:3); [α]_D_ +92 (*c* 1.0, CHCl_3_); ^1^H NMR (500 MHz, CDCl_3_) δ 5.44 (d, *J* = 10.2 Hz, 1H, NH), 4.98 (dd, *J* = 11.1, 2.9 Hz, 1H, H-3), 4.95 (d, *J* = 3.6 Hz, 1H, H-1), 4.87 (d, *J* = 12.1 Hz, 1H, CH_2(A)Troc_), 4.69–4.58 (m, 2H, H-4, CH_2(B)Troc_), 4.49 (td, *J* = 10.6, 3.6 Hz, 1H, H-2), 4.26 (dd, *J* = 12.6, 2.2 Hz, 1H, H-6_(A)_), 4.15 (dd, *J* = 12.5, 1.7 Hz, 1H, H-6_(B)_), 3.90–3.77 (m, 2H, H-5, CH_2(A)Linker_), 3.76–3.59 (m, 9H, CH_2(B)Linker_, 4 × CH_2(Linker)_), 3.39 (t, *J* = 5.1 Hz, 2H, CH_2(Linker)_), 2.07 (s, 3H, CH_3(OAc)_), 1.08 (s, 9H, C(CH_3_)_3(DTBS)_), 1.02 (s, 9H, C(CH_3_)_3(DTBS)_); ^13^C NMR (126 MHz, CDCl_3_) δ 171.3 (C=O_(OAc)_), 154.6 (C=O_(Troc)_), 98.5 (C-1), 95.8 (*C*Cl_3(Troc)_), 74.7 (CH_2(Troc)_), 71.7 (C-3), 70.9 (CH_2(Linker)_), 70.8 (CH_2(Linker)_), 70.5 (C-4), 70.27 (CH_2(Linker)_), 70.25 (CH_2(Linker)_), 67.63 (CH_2(Linker)_), 67.57 (C-5), 67.1 (C-6), 50.8 (CH_2(Linker)_), 49.3 (C-2), 27.7 (C(*C*H_3_)_3(DTBS)_), 27.4 (C(*C*H_3_)_3(DTBS)_), 23.4 (*C*(CH_3_)_3(DTBS)_), 21.1 (CH_3(OAc)_), 20.9 (*C*(CH_3_)_3(DTBS)_); HRESIMS *m*/*z*: [M + NH_4_]^+^ calcd for C_25_H_43_Cl_3_N_4_O_10_Si, 710.2158; found, 710.2158.

**2-[2-(2-Azidoethoxy)ethoxy]ethyl 2-deoxy-4,6-*O*-di-*tert*-butylsilylene-2-(2’2’2’-trichloroethoxycarbonylamino)-α-ᴅ-galactopyranoside (5):** Compound **4** (8.65 g, 12.5 mmol) was placed under N_2_ and dissolved in dry MeOH (250 mL). NaOMe (68 mg, 1.3 mmol) was added, and the reaction was stirred at room temperature for 4 hours. The solution was then neutralised with Amberlite^®^ IR120 (H^+^ form) resin, filtered and concentrated under reduced pressure. Flash chromatography on silica gel (toluene→toluene/acetone, 7:3) yielded **5** as a gold-coloured syrup (7.83 g, 96%). *R*_f_ = 0.4 (toluene/EtOAc, 3:2); [α]_D_ +67 (*c* 1.0, CHCl_3_); ^1^H NMR (500 MHz, CDCl_3_) δ 5.58 (d, *J* = 9.8 Hz, 1H, NH), 4.93 (d, *J* = 3.6 Hz, 1H, H-1), 4.77 (d, *J* = 12.0 Hz, 1H, CH_2(A)Troc_), 4.72 (d, *J* = 12.0 Hz, 1H, CH_2(B)Troc_), 4.43 (d, *J* = 3.0 Hz, 1H, H-4), 4.28 (dd, *J* = 12.5, 2.2 Hz, 1H, H-6_(A)_), 4.16 (m, 1H, H-6_(B)_), 4.11 (dd, *J* = 10.1, 3.6 Hz, 1H, H-2), 3.87–3.79 (m, 2H, H-5, CH_2(A)Linker_), 3.74 (dd, *J* = 11.4, 3.2 Hz, 1H, H-3), 3.70–3.63 (m, 9H, CH_2(B)Linker_, 4 × CH_2(Linker)_), 3.43–3.34 (m, 2H, CH_2(Linker)_), 2.53 (d, *J* = 11.8 Hz, 1H, OH), 1.07 (s, 9H, C(CH_3_)_3(DTBS)_), 1.05 (s, 9H, C(CH_3_)_3(DTBS)_); ^13^C NMR (126 MHz, CDCl_3_) δ 155.4 (C=O_(Troc)_), 98.6 (C-1), 95.7 (*C*Cl_3(Troc)_), 74.9 (CH_2(Troc)_), 73.0 (C-4), 70.9 (CH_2(Linker)_), 70.7 (CH_2(Linker)_), 70.27 (CH_2(Linker)_), 70.24 (CH_2(Linker)_), 70.14 (C-3), 67.8 (C-5), 67.7 (CH_2(Linker)_), 67.2 (C-6), 52.5 (C-2), 50.8 (CH_2(Linker)_), 27.7 (C(*C*H_3_)_3(DTBS)_), 27.5 (C(*C*H_3_)_3(DTBS)_), 23.5 (*C*(CH_3_)_3(DTBS)_), 20.9 (*C*(CH_3_)_3(DTBS)_); HRESIMS *m*/*z*: [M + Na]^+^ calcd for C_23_H_41_Cl_3_N_4_O_9_Si, 673.1606; found, 673.1605.

**2-[2-(2-Azidoethoxy)ethoxy]ethyl 2,3,4,6-tetra-*O*-acetyl-β-ᴅ-galactopyranosyl-(1→3)-2-deoxy-4,6-*O*-di-*tert*-butylsilylene-2-(2’2’2’-trichloroethoxycarbonylamino)-α-ᴅ-galactopyranoside (7):** Donor **6** [16] (7.67 g, 17.4 mmol) and acceptor **5** (7.57 g, 11.6 mmol) were placed under N_2_ together and dissolved in dry CH_2_Cl_2_ (230 mL). AW-300 4 Å molecular sieves (5.45 g) were added, and the resulting suspension was stirred at room temperature for 23 hours. NIS (5.23 g, 23.2 mmol) and AgOTf (577 mg, 2.25 mmol) were added, and the reaction was stirred at room temperature for 1 hour. Et_3_N was then added until the pH became neutral, and the suspension was filtered through Celite^®^. The filtrate was then washed with water (400 mL), brine (400 mL), dried over MgSO_4_, filtered and concentrated in vacuo. Compound **7** was isolated by flash chromatography on silica gel (toluene→toluene/EtOAc, 1:4) as an orange foam (8.97 g, 79%). *R*_f_ = 0.5 (toluene/EtOAc, 3:7); [α]_D_ +71 (*c* 1.0, CH_3_OH); ^1^H NMR (500 MHz, CD_3_OD) δ 5.40 (d, *J* = 3.4 Hz, 1H, H-4_Gal_), 5.21 (m, 1H, H-2_Gal_), 5.11 (dd, *J* = 10.4, 3.4 Hz, 1H, H-3_Gal_), 5.05 (d, *J* = 12.2 Hz, 1H, CH_2(A)Troc_), 4.93 (d, *J* = 3.6 Hz, 1H, H-1_GalNTroc_), 4.88 (d, *J* = 7.8 Hz, 1H, H-1_Gal_), 4.79 (d, *J* = 2.8 Hz, 1H, H-4_GalNTroc_), 4.58 (d, *J* = 12.2 Hz, 1H, CH_2(B)Troc_), 4.38 (dd, *J* = 11.1, 3.6 Hz, 1H, H-2_GalNTroc_), 4.30 (m, 1H, H-6_(A)_), 4.22–4.05 (m, 4H, H-5_Gal_, H-6_(B)_, H-6_(A+B)_), 3.96 (dd, *J* = 11.1, 2.8 Hz, 1H, H-3_GalNTroc_), 3.90–3.79 (m, 2H, H-5_GalNTroc_, CH_2(A)Linker_), 3.77–3.63 (m, 9H, CH_2(B)Linker_, 4 × CH_2(Linker)_), 3.46–3.39 (m, 2H, CH_2(Linker)_), 2.16 (s, 3H, CH_3(OAc)_), 2.12 (s, 3H, CH_3(OAc)_), 2.05 (s, 3H, CH_3(OAc)_), 1.97 (s, 3H, CH_3(OAc)_), 1.13–1.09 (m, 18H, 2 × C(CH_3_)_3(DTBS)_); ^13^C NMR (126 MHz, CD_3_OD) δ 171.99 (C=O_(OAc)_), 171.93 (C=O_(OAc)_), 171.6 (C=O_(OAc)_), 171.4 (C=O_(OAc)_), 156.6 (C=O_(Troc)_), 103.8 (C-1_Gal_), 99.6 (C-1_GalNTroc_), 97.2 (*C*Cl_3(Troc)_), 79.3 (C-3_GalNTroc_), 75.6 (CH_2(Troc)_), 74.1 (C-4_GalNTroc_), 72.6 (C-3_Gal_), 71.8 (C-5_Gal_), 71.52 (CH_2(Linker)_), 71.49 (CH_2(Linker)_), 71.1 (CH_2(Linker)_), 70.5 (C-2_Gal_), 69.0 (C-5_GalNTroc_), 68.6 (C-4_Gal_), 68.4 (C-6), 68.1 (CH_2(Linker)_), 62.9 (C-6), 51.8 (CH_2(Linker)_), 51.4 (C-2_GalNTroc_), 28.2 (C(*C*H_3_)_3(DTBS)_), 28.0 (C(*C*H_3_)_3(DTBS)_), 24.3 (*C*(CH_3_)_3(DTBS)_), 21.7 (*C*(CH_3_)_3(DTBS)_), 21.0 (CH_3(OAc)_), 20.6 (CH_3(OAc)_), 20.51 (CH_3(OAc)_), 20.48 (CH_3(OAc)_). HRESIMS *m*/*z*: [M + NH_4_]^+^ calcd for C_37_H_59_Cl_3_N_4_O_18_Si, 998.3003; found, 998.3003.

**2-[2-(2-Azidoethoxy)ethoxy]ethyl 2,3,4,6-tetra-*O*-acetyl-β-ᴅ-galactopyranosyl-(1→3)-2-acetamido-4,6-di-*O*-acetyl-2-deoxy-α-ᴅ-galactopyranoside** [3] **(8):** Compound **7** (8.87 g, 9.03 mmol) was placed under N_2_ and dissolved in dry THF (180 mL). 1 M Bu_4_NF/THF (32 mL, 32 mmol) was added, and the reaction was stirred at room temperature. After 2 hours, the starting material had been consumed (judged by TLC) and HF·Py (70% HF, 9.5 mL, 370 mmol) was added. Stirring was continued at room temperature for a further 3 hours and the solution was then concentrated.

The crude was placed under N_2_ and stirred at room temperature in Ac_2_O/Py (180 mL, 1:2, v/v) for 16 hours. The solution was then reduced to dryness and purification by flash chromatography on silica gel (EtOAc→EtOAc/MeOH, 17:3) yielded **8** as a dark orange/brown syrup (3.82 g, 53% over 3 steps). *R*_f_ = 0.4 (EtOAc/MeOH, 19:1); [α]_D_ +76 (*c* 1.0, CH_3_OH); ^1^H NMR (500 MHz, CD_3_OD) δ 5.42 (d, *J* = 3.1 Hz, 1H, H-4_GalNAc_), 5.36 (dd, *J* = 3.4, 1.2 Hz, 1H, H-4_Gal_), 5.06 (dd, *J* = 10.5, 3.4 Hz, 1H, H-3_Gal_), 5.00 (dd, *J* = 10.5, 7.6 Hz, 1H, H-2_Gal_), 4.83 (d, *J* = 3.8 Hz, 1H, H-1_GalNAc_), 4.78 (d, *J* = 7.6 Hz, 1H, H-1_Gal_), 4.43 (dd, *J* = 11.1, 3.6 Hz, 1H, H-2_GalNAc_), 4.26 (m, 1H, H-5_GalNAc_), 4.21–4.11 (m, 3H, H-6_(A+B)Gal_, H-6_(A)GalNAc_), 4.08 (dd, *J* = 11.1, 3.4 Hz, 1H, H-3_GalNAc_), 4.04 (m, 1H, H-5_Gal_), 3.97 (dd, *J* = 11.3, 7.3 Hz, 1H, H-6_(B)GalNAc_), 3.81 (m, 1H, CH_2(A)Linker_), 3.73–3.71 (m, 2H, CH_2(Linker)_), 3.70–3.62 (m, 7H, 3 × CH_2(Linker)_, CH_2(B)Linker_), 3.42–3.36 (m, 2H, CH_2(Linker)_), 2.14 (s, 3H, CH_3(Ac)_), 2.11 (s, 3H, CH_3(Ac)_), 2.06–2.02 (m, 9H, 3 × CH_3(Ac)_), 1.99 (s, 3H, CH_3(Ac)_), 1.93 (s, 3H, CH_3(Ac)_); ^13^C NMR (126 MHz, CD_3_OD) δ 173.1, 172.3, 172.08, 172.06, 172.04, 171.5, 171.2 (C=O_(Ac)_), 102.4 (C-1_Gal_), 99.3 (C-1_GalNAc_), 74.6 (C-3_GalNAc_), 72.2 (C-3_Gal_), 71.8 (CH_2(Linker)_), 71.54 (C-5_Gal_), 71.49 (CH_2(Linker)_), 71.36 (CH_2(Linker)_), 71.3 (CH_2(Linker)_), 71.2 (C-4_GalNAc_), 70.2 (C-2_Gal_), 68.6 (C-4_Gal_), 68.5 (C-5_GalNAc_), 68.2 (CH_2(Linker)_), 63.9 (C-6_GalNAc_), 62.4 (C-6_Gal_), 51.7 (CH_2(Linker)_), 50.3 (C-2_GalNAc_), 22.89, 20.82, 20.77, 20.73, 20.67, 20.50, 20.47 (CH_3(Ac)_). As NMR spectra in the literature were recorded in CDCl_3_ [3], NMR data are not comparable. HRESIMS *m*/*z*: [M + Na]^+^ calcd for C_32_H_48_N_4_O_19_; 815.2810; found; 815.2806.

**2-[2-(2-Azidoethoxy)ethoxy]ethyl β-ᴅ-galactopyranosyl-(1→3)-2-acetamido-2-deoxy-α-ᴅ-galactopyranoside** [3–4] **(1):** Compound **8** (1.47 g, 1.85 mmol) was dissolved in MeOH (100 mL) and freshly prepared 1 M NaOMe/MeOH was added until the solution reached pH 10. The reaction was stirred at room temperature for 1 hour, then neutralised with Amberlite^®^ IR120 (H^+^ form) resin. The resin was filtered off, washed with MeOH and the filtrate was concentrated in vacuo. After lyophilisation, **1** was obtained as a light brown/orange solid (900 mg, 90%) and required no further purification. *R*_f_ = 0.6 (EtOAc/MeOH, 2:3); [α]_D_ +76 (*c* 1.0, H_2_O); ^1^H NMR (500 MHz, D_2_O) δ 4.92 (d, *J* = 3.7 Hz, 1H, H-1_GalNAc_), 4.46 (d, *J* = 7.8 Hz, 1H, H-1_Gal_), 4.36 (dd, *J* = 11.0, 3.7 Hz, 1H, H-2_GalNAc_), 4.24 (d, *J* = 3.0 Hz, 1H, H-4_GalNAc_), 4.06 (dd, *J* = 11.1, 3.1 Hz, 1H, H-3_GalNAc_), 4.01 (m, 1H, H-5), 3.92 (d, *J* = 3.4 Hz, 1H, H-4_Gal_), 3.87 (m, 1H, CH_2(A)Linker_), 3.81–3.71 (m, 12H, 2 × H-6_(A+B)_, 4 × CH_2(Linker)_), 3.70–3.59 (m, 3H, H-3_Gal_, H-5, CH_2(B)Linker_), 3.56–3.48 (m, 3H, H-2_Gal_, CH_2(Linker)_), 2.04 (s, 3H, CH_3(NHAc)_); ^13^C NMR (126 MHz, D_2_O) δ 174.5 (C=O_(NHAc)_), 104.7 (C-1_Gal_), 97.4 (C-1_GalNAc_), 77.3 (C-3_GalNAc_), 74.9 (C-5), 72.5 (C-3_Gal_), 70.64 (C-5), 70.54 (C-2_Gal_), 69.7 (CH_2(Linker)_), 69.51 (CH_2(Linker)_), 69.46 (CH_2(Linker)_), 69.2 (CH_2(Linker)_), 68.7 (C-4_GalNAc_), 68.5 (C-4_Gal_), 66.5 (CH_2(Linker)_), 61.1 (C-6), 60.9 (C-6), 50.1 (CH_2(Linker)_), 48.5 (C-2_GalNAc_), 22.0 (CH_3(NHAc)_). NMR data match those reported in the literature [3–4]. HRESIMS *m*/*z*: [M + H]^+^ calcd for C_20_H_36_N_4_O_13_, 541.2357; found, 541.2354.

**2-[2-(2-Azidoethoxy)ethoxy]ethyl 3-*O*-sulfo-β-ᴅ-galactopyranosyl-(1→3)-2-acetamido-2-deoxy-α-ᴅ-galactopyranoside sodium salt (2):** Compound **1** (1.24 g, 2.29 mmol) and Bu_2_SnO (645 mg, 2.75 mmol) were placed under N_2_ together. Dry benzene/DMF (380 mL, 5:1, v/v) was added and the reaction was refluxed at 125 °C using a Dean–Stark apparatus. After 24 hours, the solvent in the receiver was drained, and the benzene was removed from the reaction mixture in vacuo. SO_3_·NMe_3_ (642 mg, 4.61 mmol) was then added to the DMF solution, and the reaction was stirred at room temperature. After 24 hours, an additional portion of SO_3_·NMe_3_ (950 mg, 6.83 mmol) was added and stirring was continued at room temperature for a further 48 hours. The reaction mixture was then concentrated and flash chromatography on silica gel (EtOAc/MeOH, 1:0→0:1) yielded a yellow syrup, which was re-dissolved in H_2_O (30 mL). Dowex^®^ 50WX4 (Na^+^ form) resin (1.28 g) was added, and the resulting suspension was stirred at room temperature for 16 hours. Filtration followed by concentration and lyophilisation of the filtrate yielded **2** as a pale-yellow foam (972 mg, 66%). *R*_f_ = 0.3 (EtOAc/MeOH, 3:2); [α]_D_ +76 (*c* 1.0, H_2_O); ^1^H NMR (500 MHz, D_2_O) δ 4.95 (d, *J* = 3.8 Hz, 1H, H-1_GalNAc_), 4.62 (d, *J* = 7.9 Hz, 1H, H-1_Gal_), 4.41–4.31 (m, 4H, H-2_GalNAc_, H-3_Gal_, H-4_Gal_, H-4_GalNAc_), 4.30–4.16 (m, 5H, H-5, 2 × H-6_(A+B)_), 4.11 (dd, *J* = 11.1, 3.1 Hz, 1H, H-3_GalNAc_), 3.97 (t, *J* = 6.2 Hz, 1H, H-5), 3.91 (m, 1H, CH_2(A)Linker_), 3.85–3.62 (m, 10H, H-2_Gal_, CH_2(B)Linker_, 4 × CH_2(Linker)_), 3.56–3.50 (m, 2H, CH_2(Linker)_), 2.05 (s, 3H, CH_3(NHAc)_); ^13^C NMR (126 MHz, D_2_O) δ 174.5 (C=O_(NHAc)_), 104.2 (C-1_Gal_), 97.4 (C-1_GalNAc_), 79.9 (C-3_Gal_), 77.5 (C-3_GalNAc_), 72.1 (C-5), 69.6 (CH_2(Linker)_), 69.47 (CH_2(Linker)_), 69.38 (CH_2(Linker)_), 69.2 (CH_2(Linker)_), 68.8 (C-5), 68.55 (C-2_Gal_), 68.52 (C-4_GalNAc_), 68.3 (C-6), 66.9 (C-6), 66.7 (CH_2(Linker)_), 66.5 (C-4_Gal_), 50.1 (CH_2(Linker)_), 48.3 (C-2_GalNAc_), 22.0 (CH_3(NHAc)_). HRESIMS *m*/*z*: [M – Na + 2H]^+^ calcd for C_20_H_37_N_4_O_16_S, 621.1925; found, 621.1920.

## Supporting Information

File 1 NMR spectra of compounds **1**–**5**, **7** and **8**.
