# Supplementary material for: Synthesis of the 3’-O-sulfated TF antigen with a TEG-N3 linker for glycodendrimersomes preparation to study lectin binding
Source: Beilstein J Org Chem. 2024 Jan 30;20:173–80. doi: 10.3762/bjoc.20.17 (PMC10840529; doi:10.3762/bjoc.20.17)

BEILSTEIN JOURNAL OF ORGANIC CHEMISTRY

Supporting Information

for

Synthesis of the 3’-*O*-sulfated TF antigen with a TEG-N_3 linker for glycodendrimersomes preparation to study lectin binding

Mark Reihill, Hanyue Ma, Dennis Bengtsson and Stefan Oscarson

Beilstein J. Org. Chem. **2024,** *20,* 173–180. doi:10.3762/bjoc.20.17

NMR spectra of compounds 1–5, 7 and 8

License and Terms: This is a supporting information file under the terms of the Creative Commons Attribution License (https://creativecommons.org/licenses/by/4.0). Please note that the reuse, redistribution and reproduction in particular requires that the author(s) and source are credited and that individual graphics may be subject to special legal provisions.
The license is subject to the *Beilstein Journal of Organic Chemistry* terms and conditions: (https://www.beilstein-journals.org/bjoc/terms)

Table of content

Phenyl 3-*O*-acetyl-2-deoxy-4,6-*O*-di-*tert*-butylsilylene-1-thio-2-(2’2’2’-trichloroethoxycarbonylamino)-β-D-galactopyranoside[9,10] (**3**)
^{1}H NMR, 500 MHz……S2
^{13}C NMR, 126 MHz……S3
2-[2-(2-Azidoethoxy)ethoxy]ethyl 3-*O*-acetyl-2-deoxy-4,6-*O*-di-*tert*-butylsilylene-2-(2’2’2’-trichloroethoxycarbonylamino)-α-D-galactopyranoside (**4**)
^{1}H NMR, 500 MHz……S4
^{13}C NMR, 126 MHz……S5
2-[2-(2-Azidoethoxy)ethoxy]ethyl 2-deoxy-4,6-*O*-di-*tert*-butylsilylene-2-(2’2’2’-trichloroethoxycarbonylamino)-α-D-galactopyranoside (**5**)
^{1}H NMR, 500 MHz……S6
^{13}C NMR, 126 MHz……S7
2-[2-(2-Azidoethoxy)ethoxy]ethyl 2,3,4,6-tetra-*O*-acetyl-β-D-galactopyranosyl-(1→3)-2-deoxy-4,6-*O*-di-*tert*-butylsilylene-2-(2’2’2’-trichloroethoxycarbonylamino)-α-D-galactopyranoside (**7**)
^{1}H NMR, 500 MHz……S8
2-[2-(2-Azidoethoxy)ethoxy]ethyl 2,3,4,6-tetra-*O*-acetyl-β-D-galactopyranosyl-(1→3)-2-acetamido-4,6-di-*O*-acetyl-2-deoxy-α-D-galactopyranoside[3] (**8**)
^{1}H NMR, 500 MHz……S9
^{13}C NMR, 126 MHz……S10
2-[2-(2-Azidoethoxy)ethoxy]ethyl β-D-galactopyranosyl-(1→3)-2-acetamido-2-deoxy-α-D-galactopyranoside[3,4] (**1**)
^{1}H NMR, 500 MHz……S11
HSQC, ^{1}H NMR, 500 MHz, ^{13}C NMR……S12
2-[2-(2-Azidoethoxy)ethoxy]ethyl 3-*O*-sulfo-β-D-galactopyranosyl-(1→3)-2-acetamido-2-deoxy-α-D-galactopyranoside sodium salt (**2**)
^{1}H NMR, 500 MHz……S13
^{13}C NMR, 126 MHz……S14
HSQC, ^{1}H NMR, 400 MHz, ^{13}C NMR……S15

Phenyl 3-*O*-acetyl-2-deoxy-4,6-*O*-di-*tert*-butylsilylene-1-thio-2-(2'2'2'-trichloroethoxycarbonylamino)-β-D-galactopyranoside[9,10] (3)

^{1}H NMR, 500 MHz

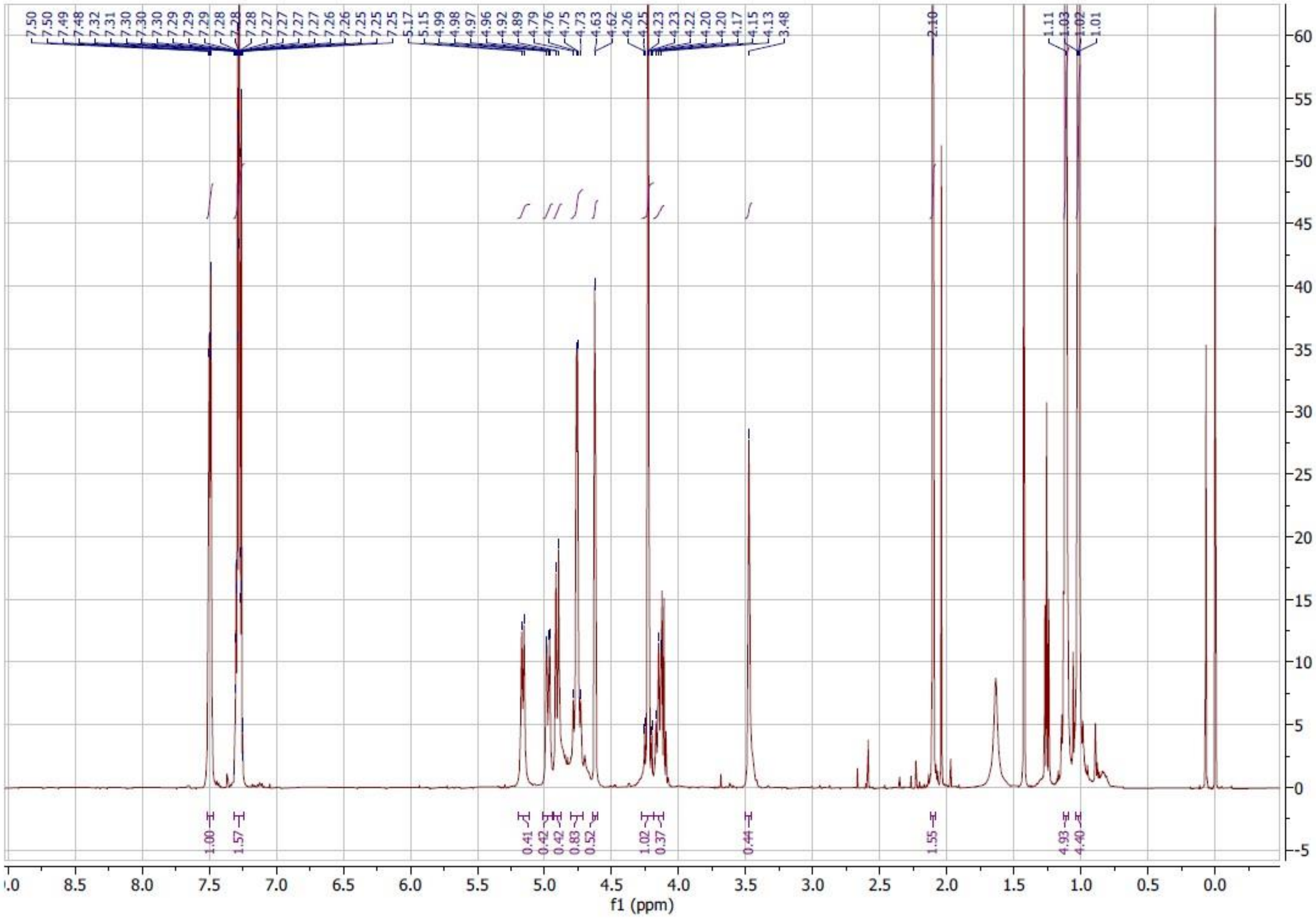

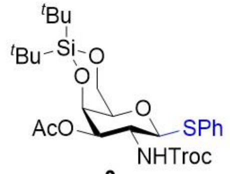

3

Phenyl 3-*O*-acetyl-2-deoxy-4,6-*O*-di-*tert*-butylsilylene-1-thio-2-(2'2'2'-trichloroethoxycarbonylamino)-β-D-galactopyranoside[9,10] (3)

^{13}C NMR, 126 MHz

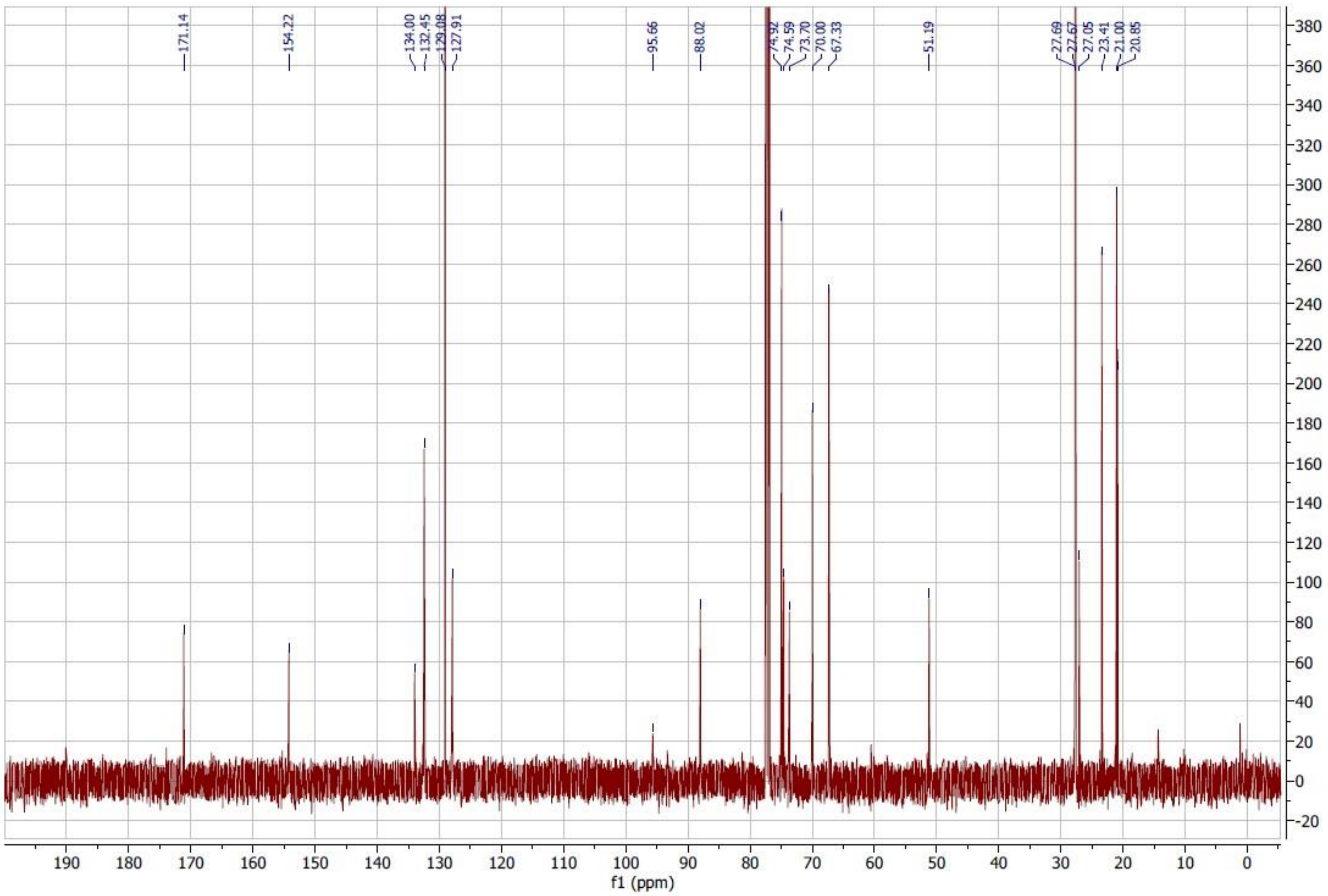

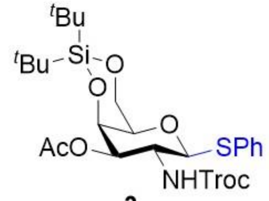

3

2-[2-(2-Azidoethoxy)ethoxy]ethyl 3-*O*-acetyl-2-deoxy-4,6-*O*-di-*tert*-butylsilylene-2-(2'2'2'-trichloroethoxycarbonylamino)-α-D-galactopyranoside (4)

^{1}H NMR, 500 MHz

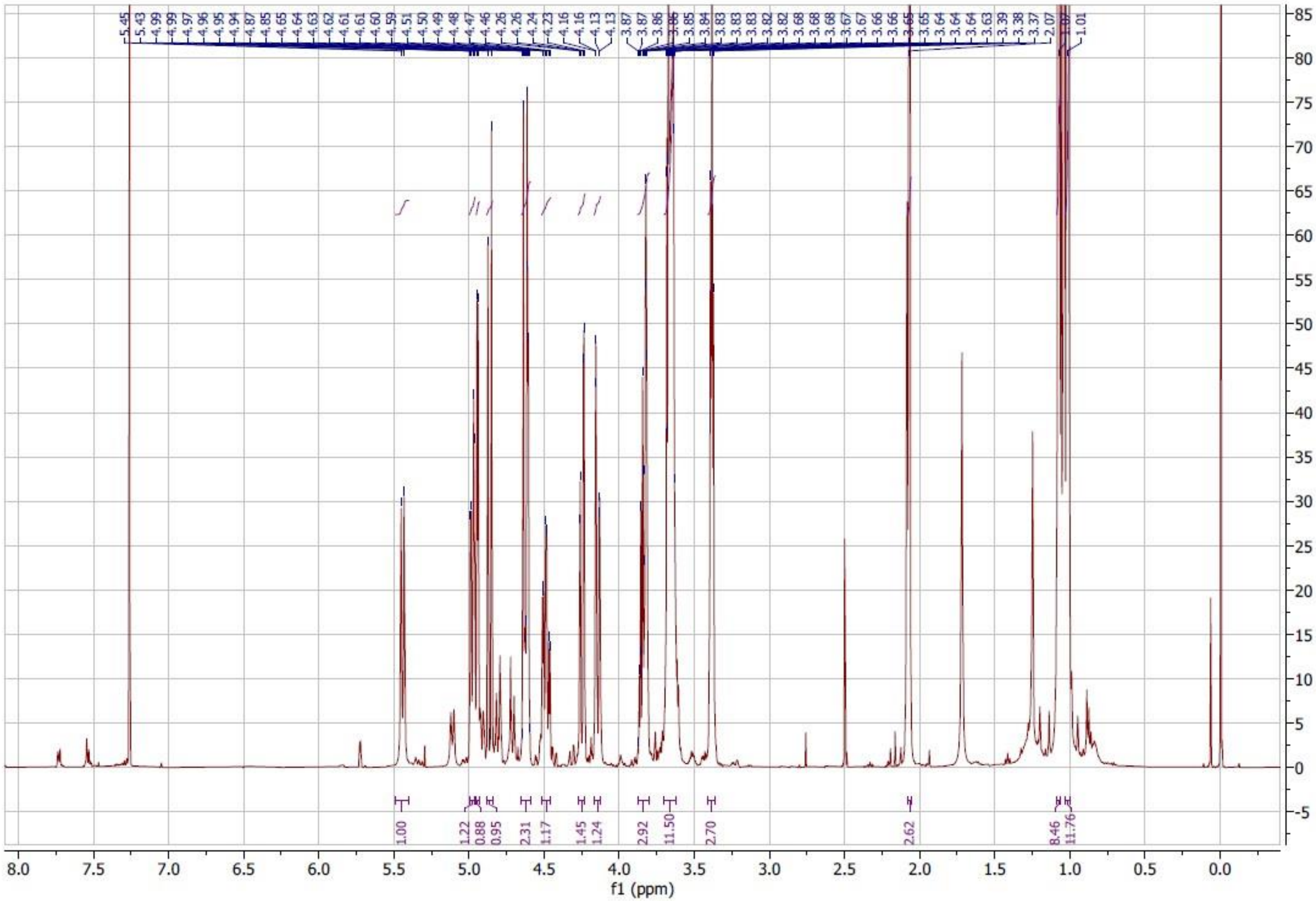

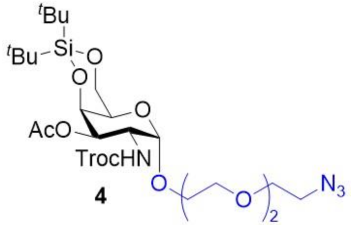

2-[2-(2-Azidoethoxy)ethoxy]ethyl 3-*O*-acetyl-2-deoxy-4,6-*O*-di-*tert*-butylsilylene-2-(2'2'2'-trichloroethoxycarbonylamino)-α-D-galactopyranoside (4)

^{13}C NMR, 126 MHz

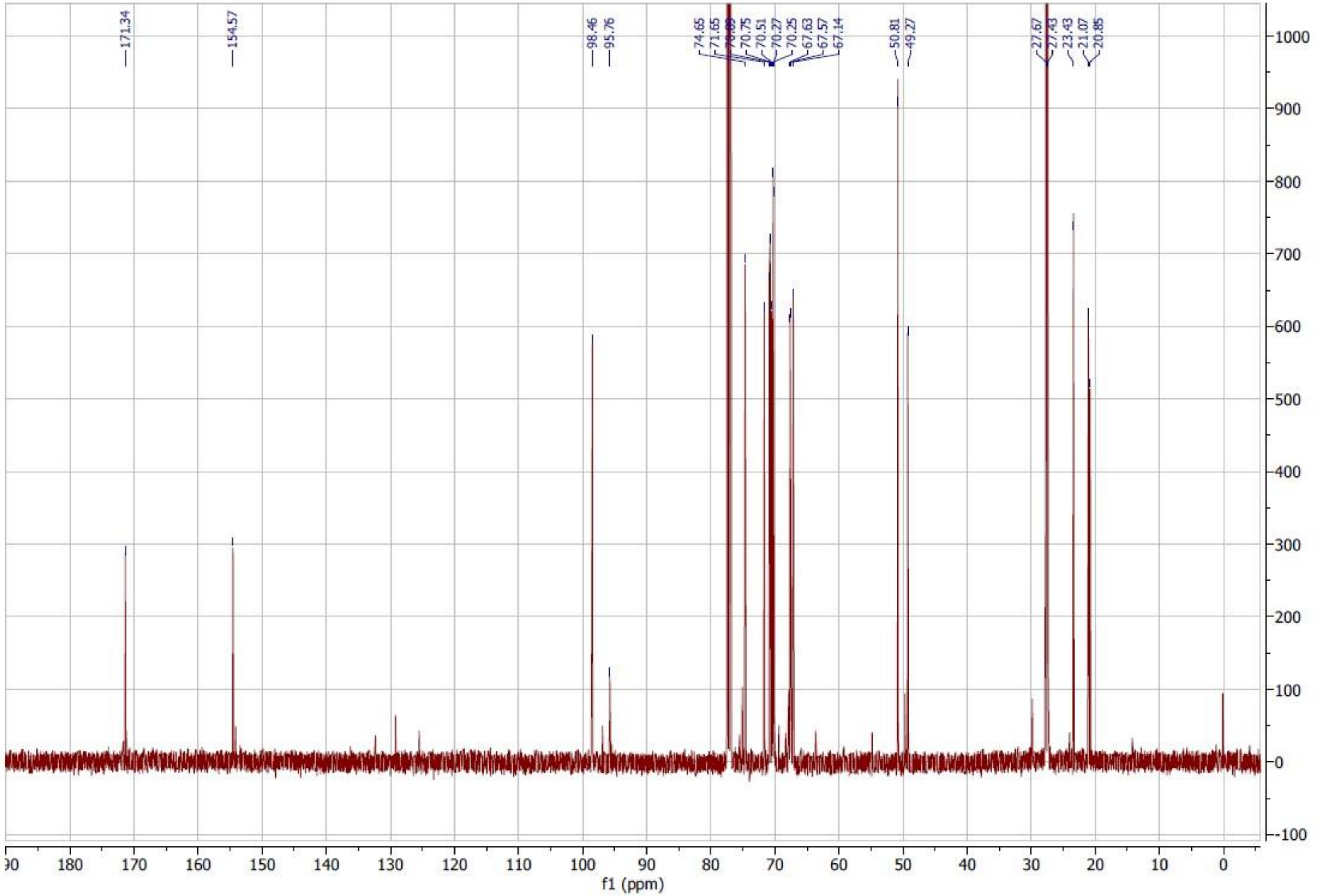

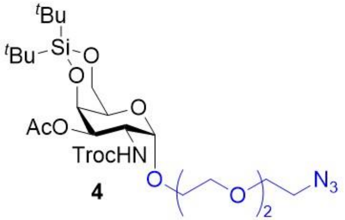

4

2-[2-(2-Azidoethoxy)ethoxy]ethyl 2-deoxy-4,6-*O*-di-*tert*-butylsilylene-2-(2'2'2'-trichloroethoxycarbonylamino)-α-D-galactopyranoside (5)

^{1}H NMR, 500 MHz

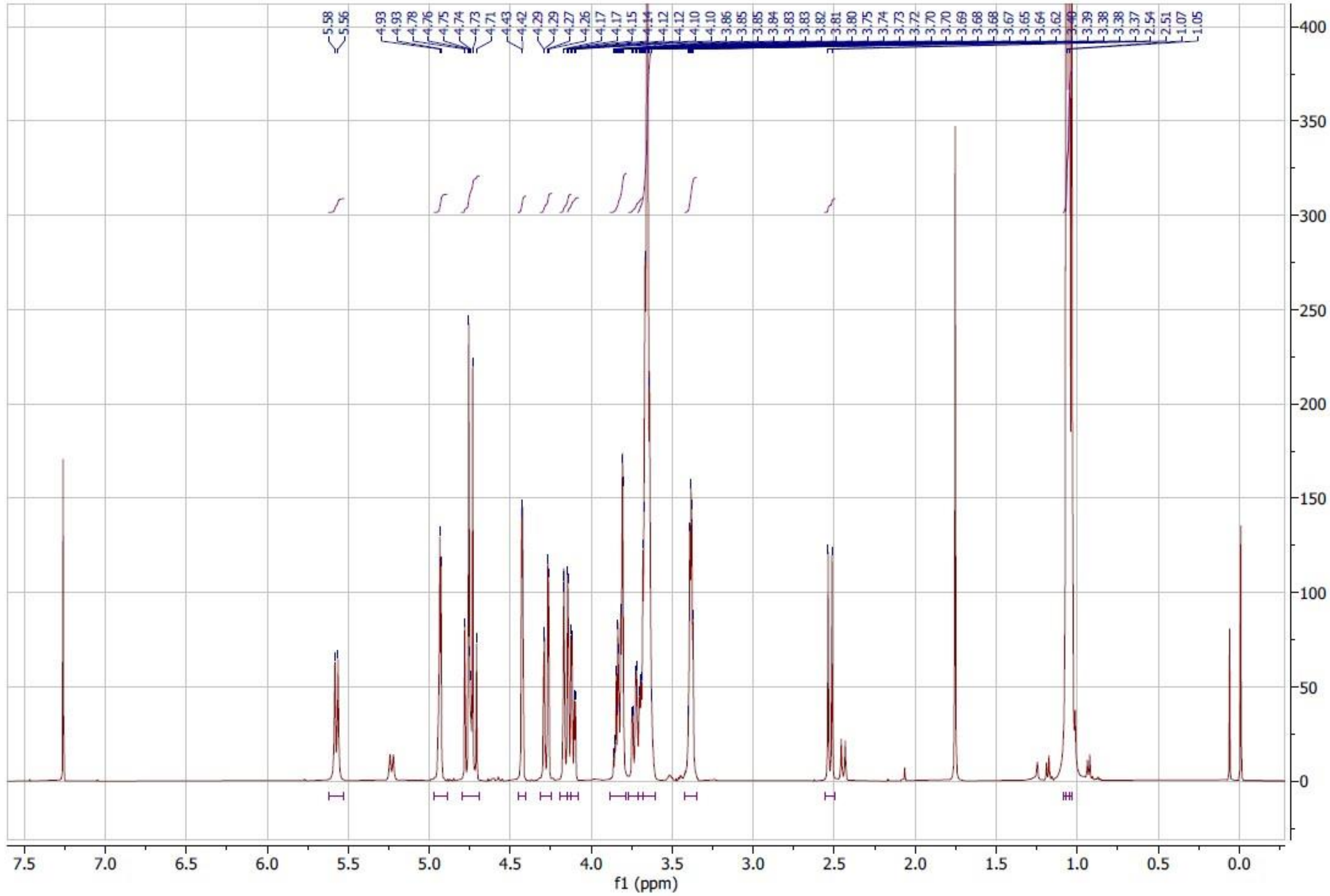

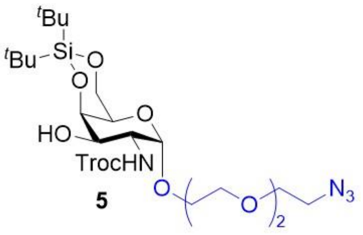

5

2-[2-(2-Azidoethoxy)ethoxy]ethyl 2-deoxy-4,6-*O*-di-*tert*-butylsilylene-2-(2'2'2'-trichloroethoxycarbonylamino)-α-D-galactopyranoside (5)

^{13}C NMR, 126 MHz

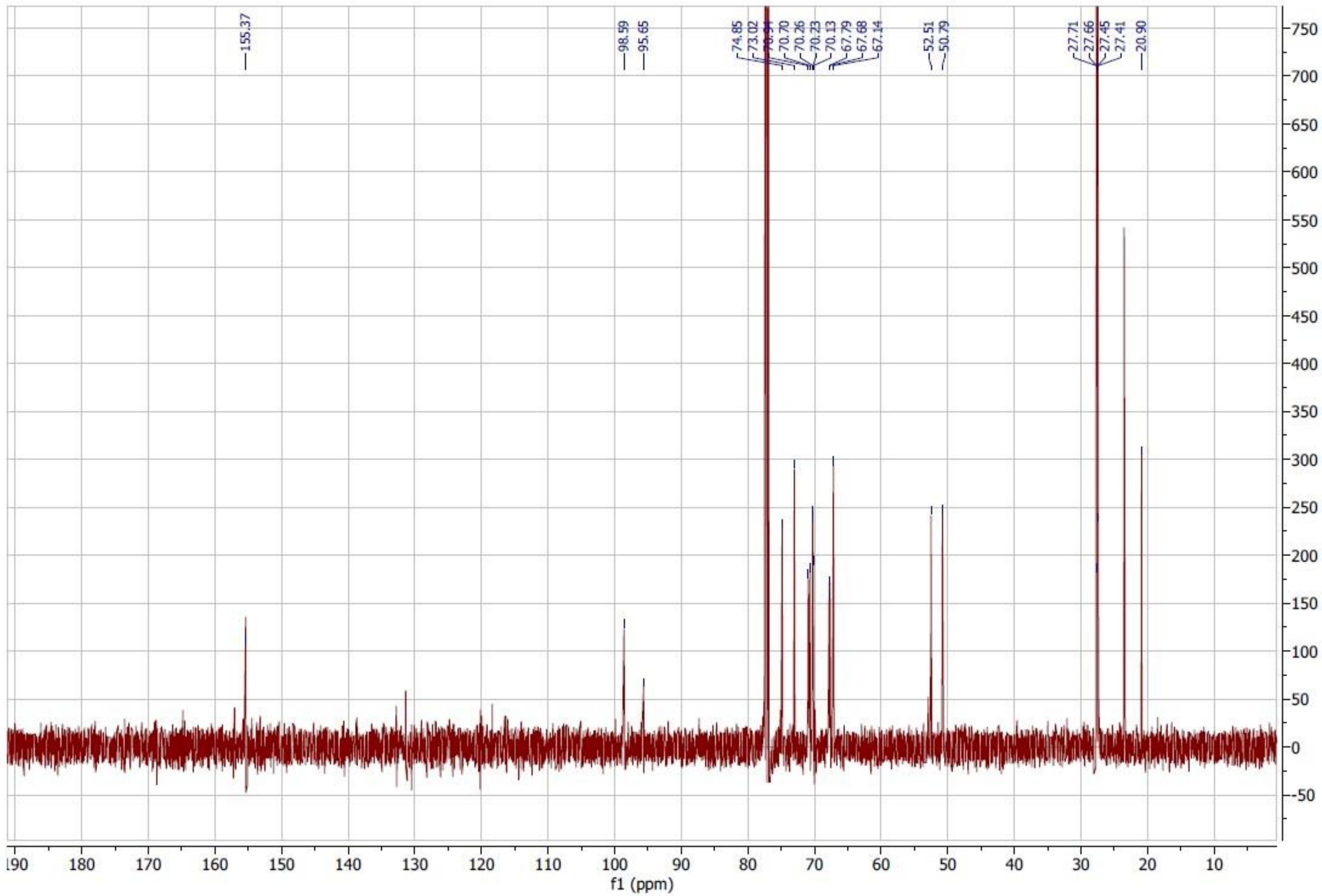

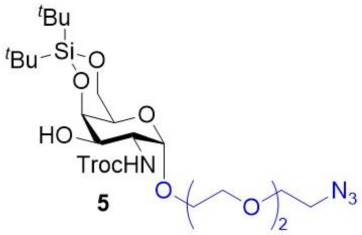

5

2-[2-(2-Azidoethoxy)ethoxy]ethyl 2,3,4,6-tetra-*O*-acetyl-β-D-galactopyranosyl-(1→3)-2-deoxy-4,6-*O*-di-*tert*-butylsilylene-2-(2'2'2'-trichloroethoxycarbonylamino)-α-D-galactopyranoside (7)

^{1}H NMR, 500 MHz

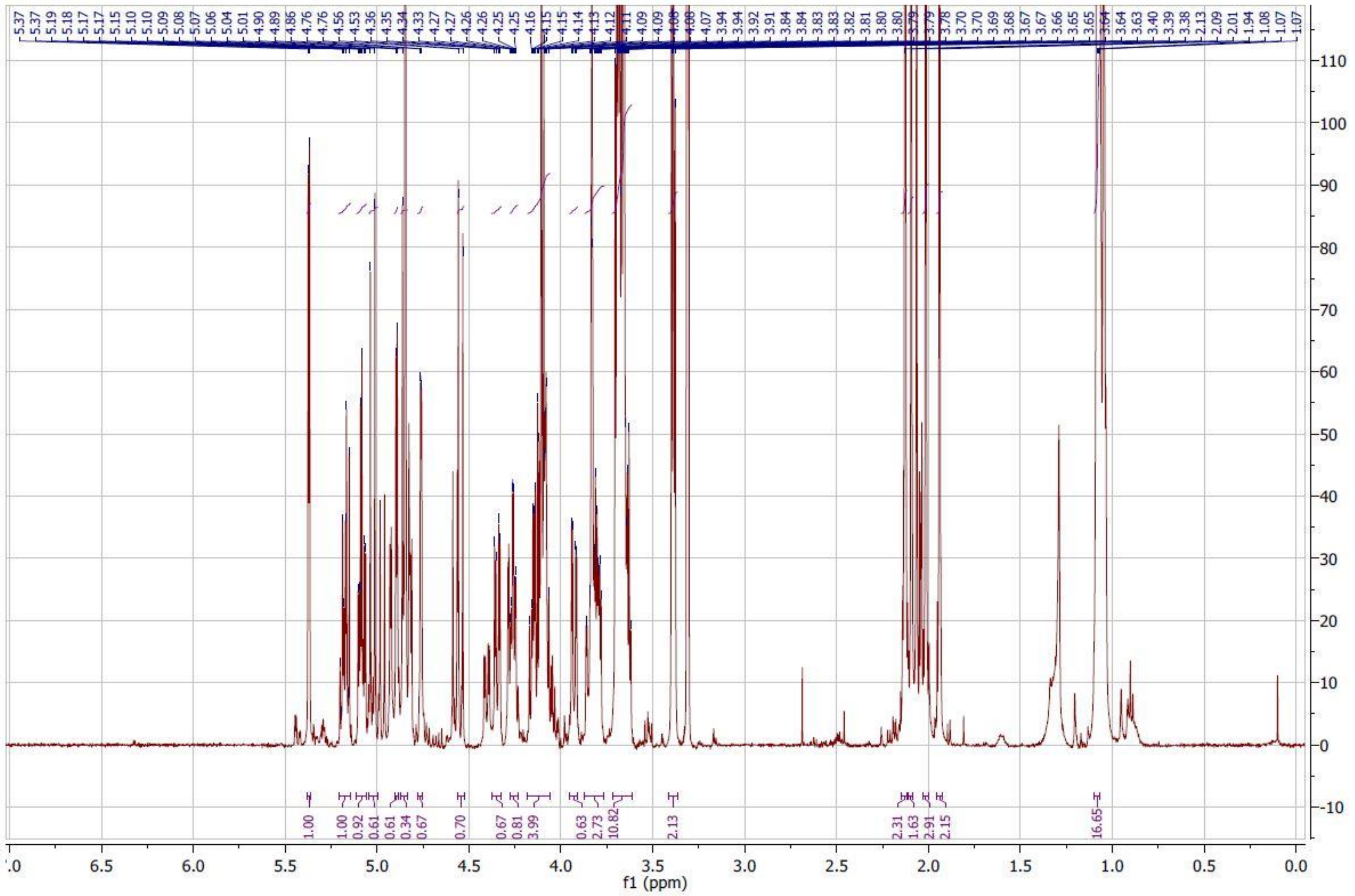

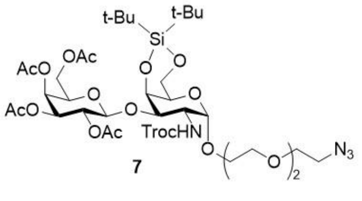

2-[2-(2-Azidoethoxy)ethoxy]ethyl 2,3,4,6-tetra-*O*-acetyl-β-D-galactopyranosyl-(1→3)-2-acetamido-4,6-di-*O*-acetyl-2-deoxy-α-D-galactopyranoside[3] (8)

^{1}H NMR, 500 MHz

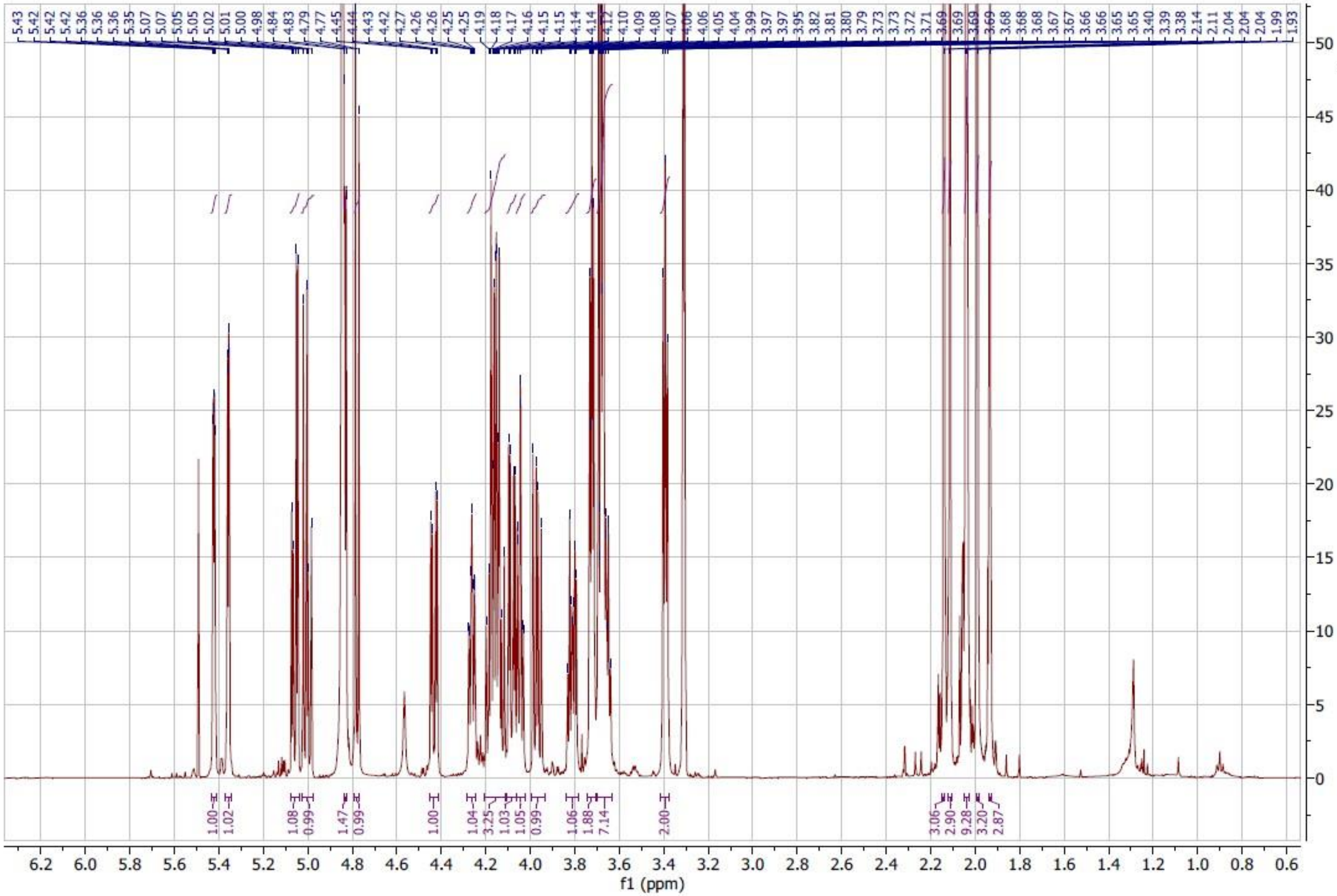

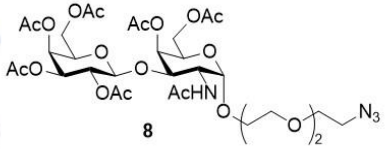

8

2-[2-(2-Azidoethoxy)ethoxy]ethyl 2,3,4,6-tetra-*O*-acetyl-β-D-galactopyranosyl-(1→3)-2-acetamido-4,6-di-*O*-acetyl-2-deoxy-α-D-galactopyranoside[3] (8)

^{13}C NMR, 126 MHz

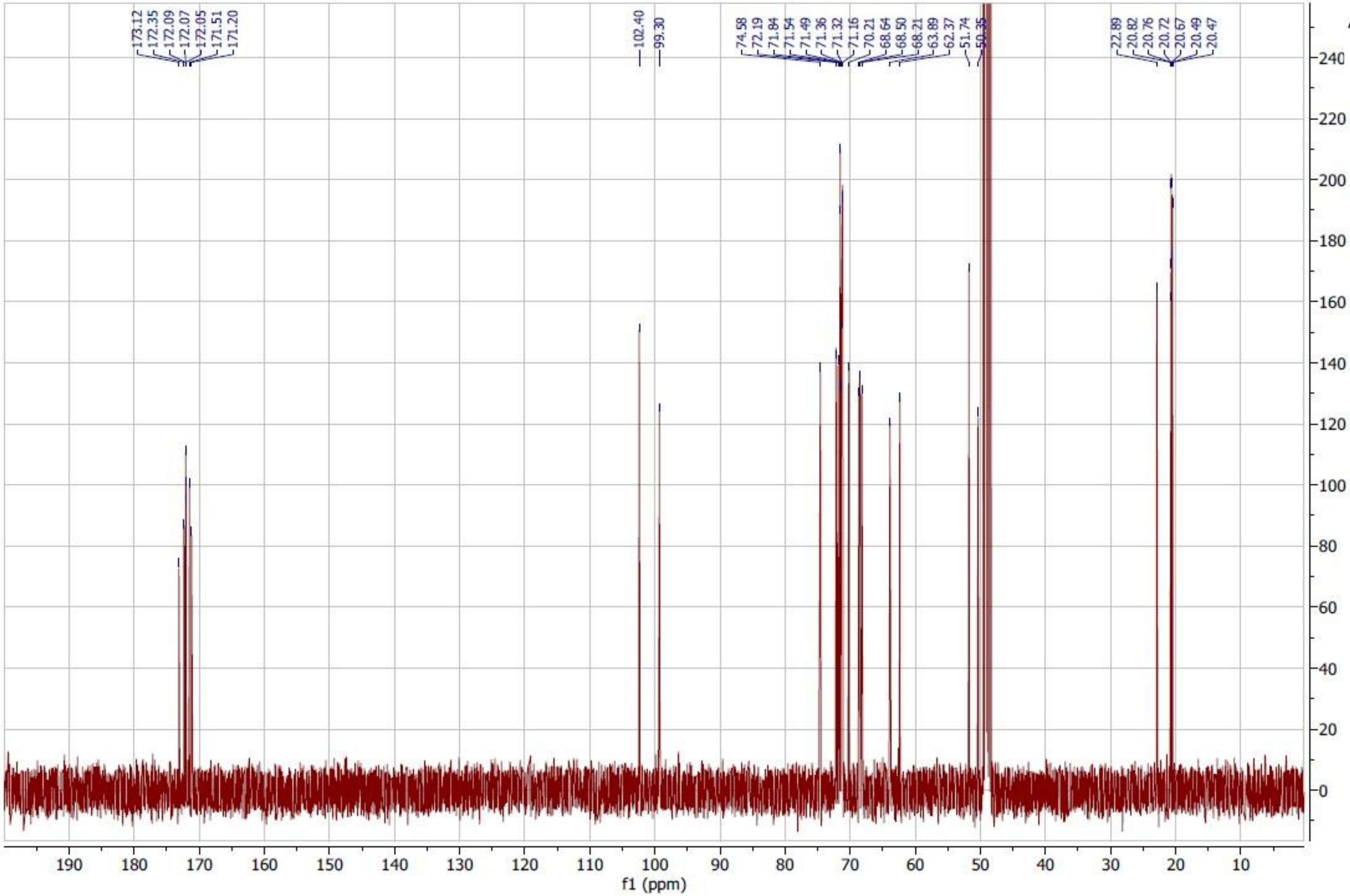

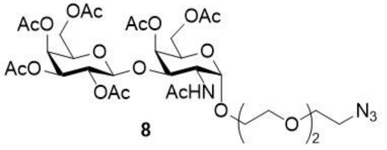

2-[2-(2-Azidoethoxy)ethoxy]ethyl β-D-galactopyranosyl-(1→3)-2-acetamido-2-deoxy-α-D-galactopyranoside[3,4] (1)

^{1}H NMR, 500 MHz

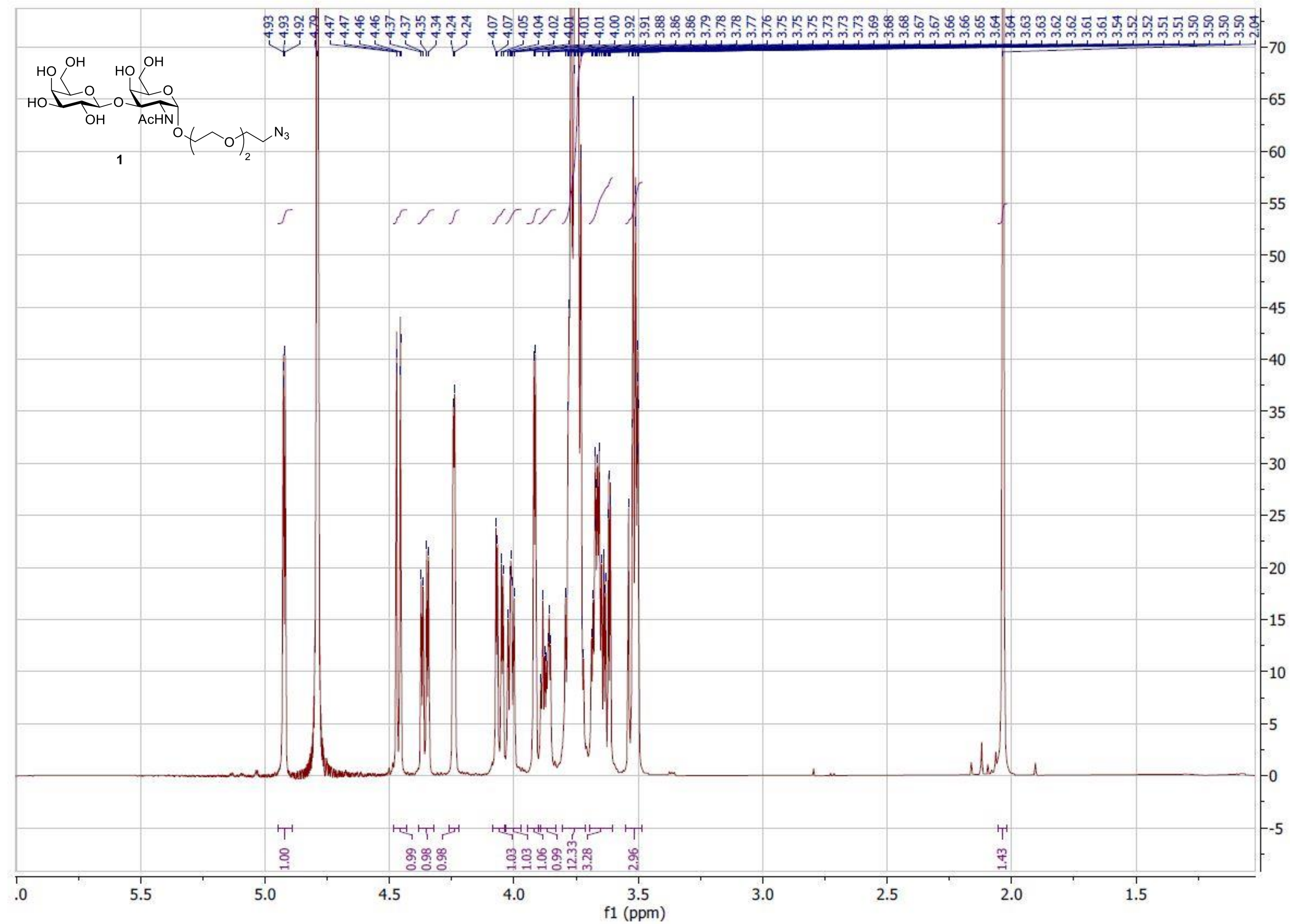

2-[2-(2-Azidoethoxy)ethoxy]ethyl β-D-galactopyranosyl-(1→3)-2-acetamido-2-deoxy-α-D-galactopyranoside[3,4] (1)

HSQC, ^{1}H NMR, 500 MHz, ^{13}C NMR projected

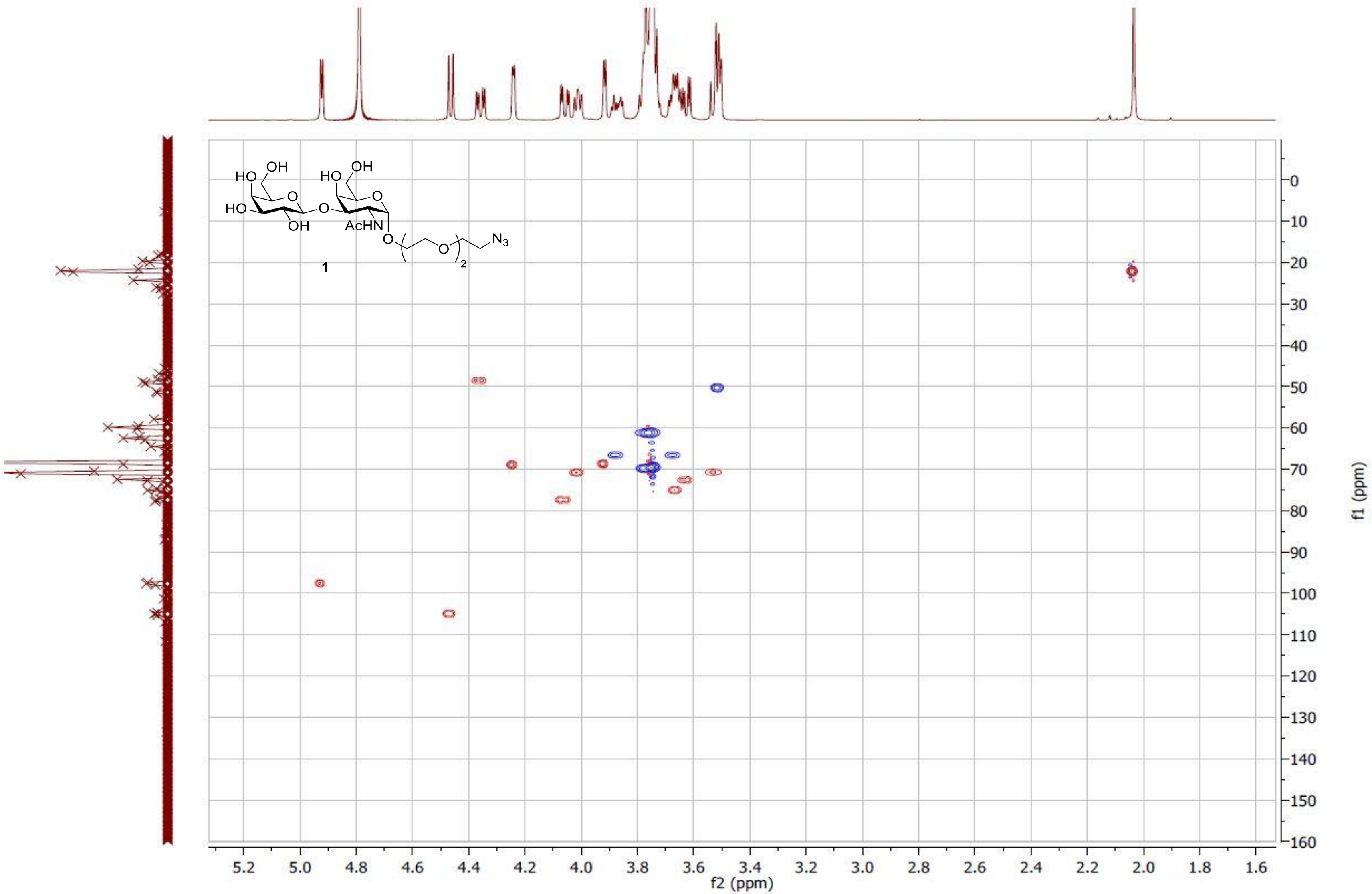

2-[2-(2-Azidoethoxy)ethoxy]ethyl 3-*O*-sulfo-β-D-galactopyranosyl-(1→3)-2-acetamido-2-deoxy-α-D-galactopyranoside sodium salt (2)

^{1}H NMR, 400 MHz

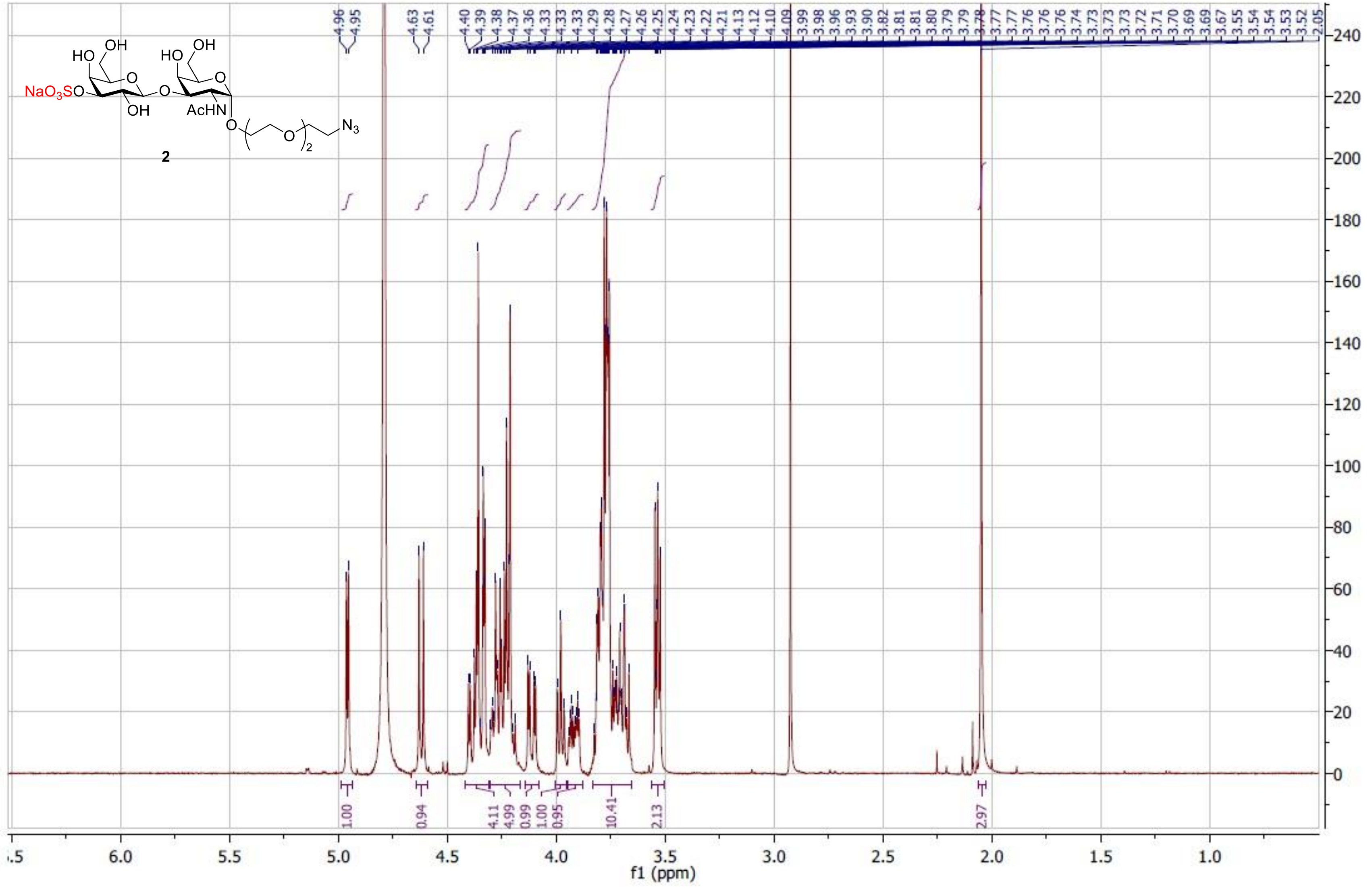

2-[2-(2-Azidoethoxy)ethoxy]ethyl 3-*O*-sulfo-β-D-galactopyranosyl-(1→3)-2-acetamido-2-deoxy-α-D-galactopyranoside sodium salt (2)

^{13}C NMR, 101 MHz

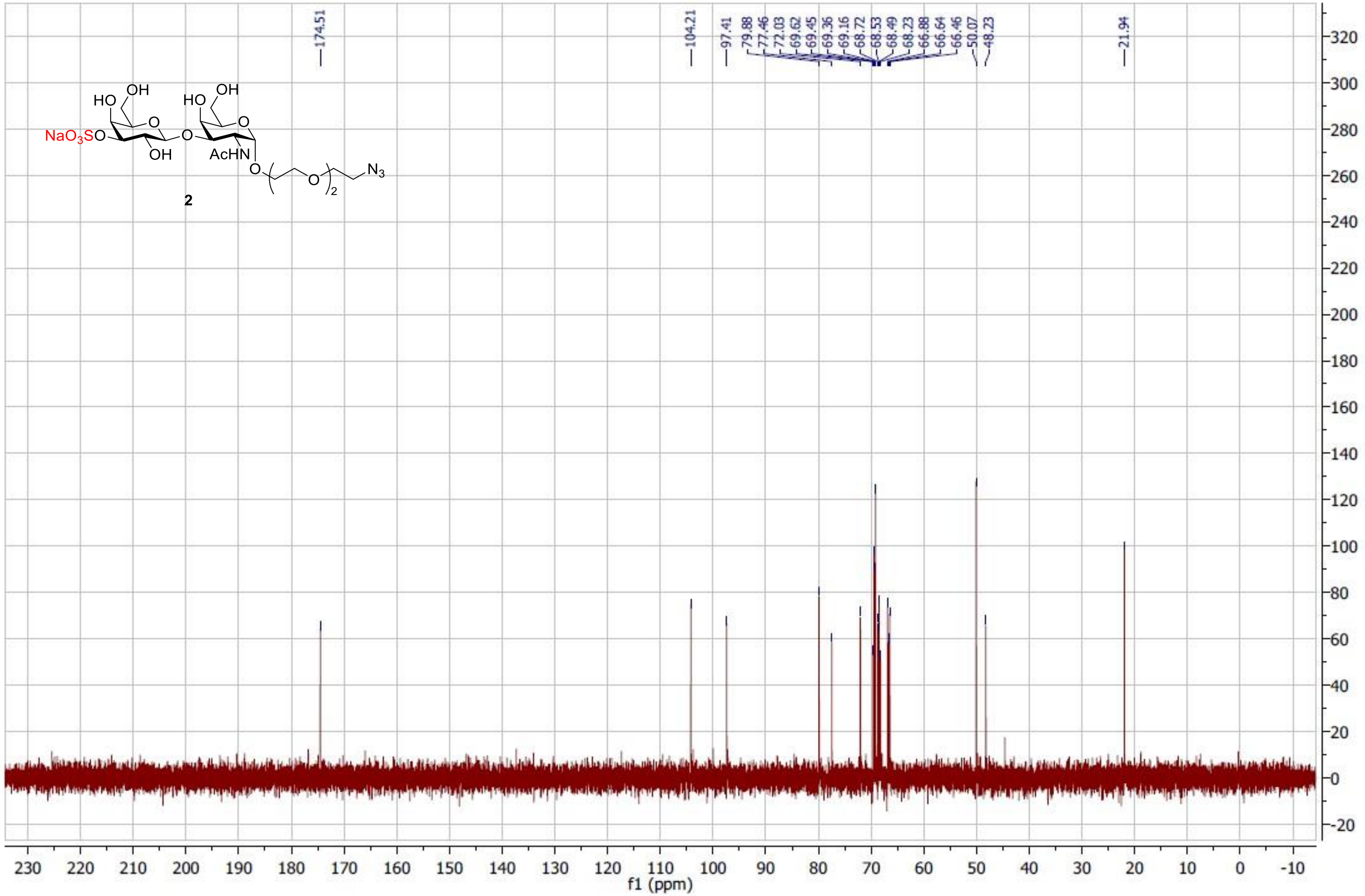

2-[2-(2-Azidoethoxy)ethoxy]ethyl 3-*O*-sulfo-β-D-galactopyranosyl-(1→3)-2-acetamido-2-deoxy-α-D-galactopyranoside sodium salt (2)

HSQC, ^{1}H NMR, 400 MHz, ^{13}C NMR, 101 MHz

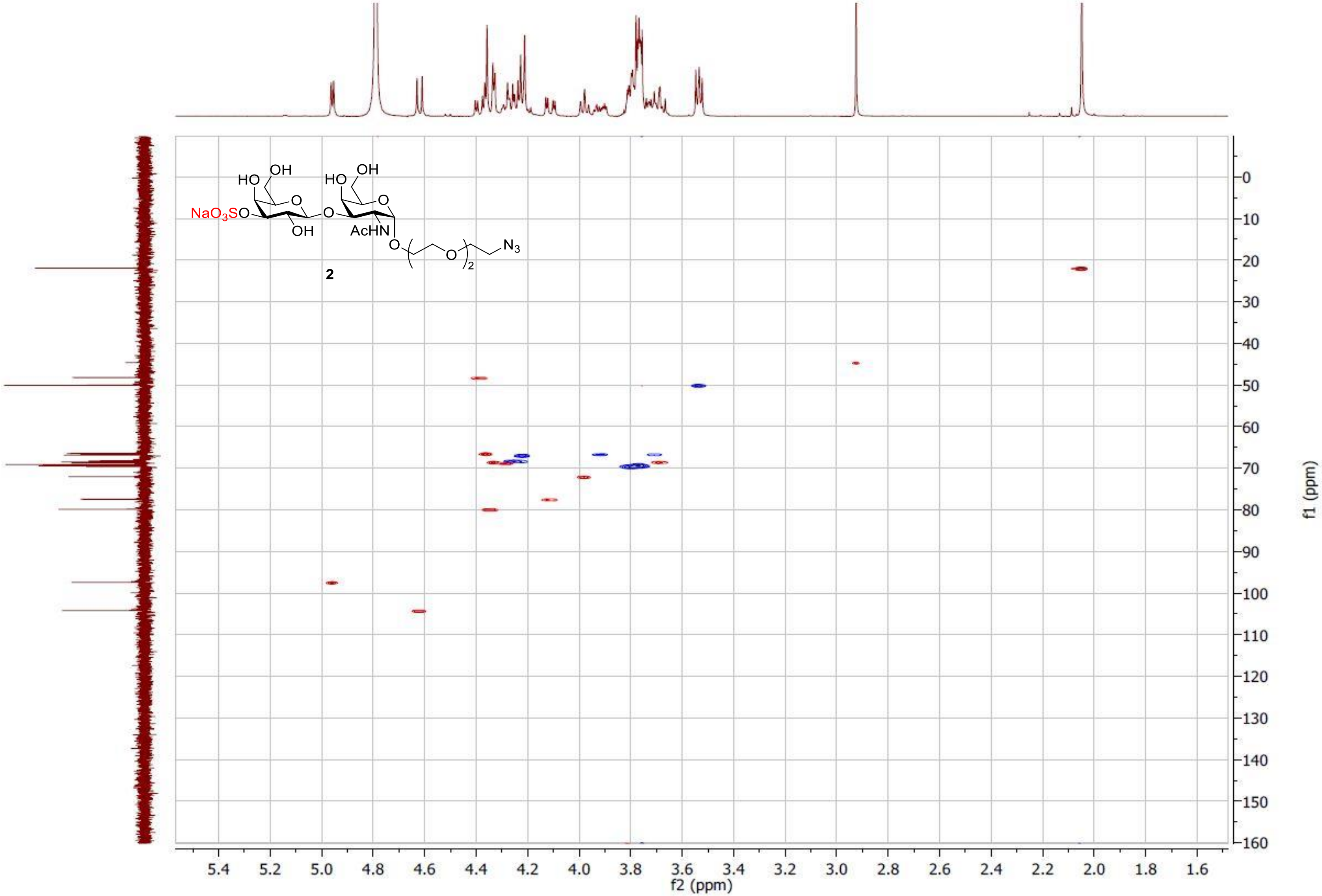

Supplement: File 1 — NMR spectra of compounds 1–5, 7 and 8. [file Beilstein_J_Org_Chem-20-173-s001.pdf]
